# Association of diabetic retinopathy on all-cause and cause-specific mortality in older adults with diabetes: National Health and Nutrition Examination Survey, 2005–2008

**DOI:** 10.1038/s41598-024-58502-z

**Published:** 2024-05-07

**Authors:** Kun Liang, Siyu Gui, Xinchen Wang, Qianqian Wang, Jianchao Qiao, Liming Tao, Heting Liu, Zhengxuan Jiang, Jie Gao

**Affiliations:** 1 Department of Ophthalmology, The Second Affiliated Hospital of Anhui Medical University, 678 Furong Road, Hefei, 230601 China; 2 Department of Clinical Medicine, The Second School of Clinical Medicine, Anhui Medical University, 81 Meishan Road, Hefei, 230032 China

**Keywords:** Diabetic retinopathy, Mortality, Cardiovascular disease, Diabetes mellitus, Propensity score matching, Environmental social sciences, Endocrinology, Risk factors, Signs and symptoms

## Abstract

To evaluate the effect of diabetic retinopathy (DR) status or severity on all-cause and cause-specific mortality among diabetic older adults in the United States using the most recent National Health and Nutrition Examination Survey (NHANES) follow-up mortality data. The severity of DR was graded according to the Early Treatment Diabetic Retinopathy Study (ETDRS) grading scale. Multiple covariate-adjusted Cox proportional hazards regression models, Fine and Gray competing risk regression models, and propensity score matching (PSM) methods were used to assess the risk of all-cause and cause-specific mortality in individuals with diabetes. All analyses adopted the weighted data and complex stratified design approach proposed by the NHANES guidelines. Time to death was calculated based on the time between baseline and date of death or December 31, 2019, whichever came first. Ultimately 1077 participants, representing 3,025,316 US non-hospitalized individuals with diabetes, were included in the final analysis. After a median follow-up of 12.24 years (IQR, 11.16–13.49), 379 participants were considered deceased from all-causes, with 43.90% suffering from DR, including mild DR (41.50%), moderate to severe DR (46.77%), and proliferative DR (PDR) (67.21%). DR was associated with increased all-cause, cardiovascular disease (CVD) and diabetes mellitus (DM)-specific mortality, which remained consistent after propensity score matching (PSM). Results of DR grading assessment suggested that the presence of mild, moderate to severe NPDR was significantly associated with increased risk of all-cause and CVD-specific mortality, while the presence and severity of any DR was associated with increased DM-specific mortality, with a positive trend. The presence of DR in elderly individuals with diabetes is significantly associated with the elevated all-cause and CVD mortality. The grading or severity of DR may reflect the severity of cardiovascular disease status and overall mortality risk in patients with diabetes.

## Introduction

Diabetic retinopathy (DR) is the most common form of microvascular disease and the most common type of retinal vascular disease. As a microvascular complication of diabetes mellitus (DM), almost all type 1 diabetes (T1D) patients and over 60% of type 2 diabetes (T2D) patients have a significant risk of developing DR after 10 years of diabetes^1^. The progression of the disease to the macula can lead to significant vision loss and eventual blindness, making it a major cause of blindness in individuals with diabetes^2^. DR is often accompanied by ocular comorbidities such as ischemic optic neuropathy caused by hyperglycemia, cataracts caused by lens opacity, and neovascular glaucoma caused by changes in aqueous humor osmotic pressure^3^. DR is generally classified into non-proliferative and proliferative stages^4^. Owing to enhanced clinical management and improved treatments in recent years, the long-term prognosis of individuals with diabetes has improved to a certain extent. However, their mortality rate is still higher than that of the general population, with type 2 diabetes and its related complications accounting for 8.4% of global deaths^5^, consuming many medical resources. Many studies have shown that large vessel disease is the main contributor to mortality in individuals with diabetes, but the role of microvascular complications, especially the dynamic development of DR, in mortality risk is still unclear^6^. On the other hand, DR patients have been shown to be in a subclinical state of cardiovascular disease (CVD), making it difficult to detect early signs of CVD that affect mortality risk through routine examinations. This emphasizes the need for standardized examinations and assessments to identify new individuals with diabetes with cardiovascular disease and mortality risk^7^.

DR is the main cause of vision loss in the United States, as well as the main preventable cause of blindness in working-age people. The International Diabetes Federation (IDF) estimates that the number of people with diabetes worldwide will reach 463 million by 2019 and 700 million by 2045^2^. Despite the enormous burden that DR imposes on public health systems, the basic mechanisms underlying its occurrence and development in DM patients are still poorly understood. Previous epidemiological studies have reported that DR is associated with some complications such as cardiovascular disease^8^. As the only part of the body where blood vessels can be directly observed, the examination of the fundus and imaging of the retina have significant potential for the convenient and rapid assessment of the overall burden of vascular or systemic diseases in DR patients. However, the relationship between diabetic retinopathy and survival rates is still contradictory. This may be related to the inadequate adjustment of important confounding factors, such as systemic comorbidities (such as cardiovascular complications, cancer, etc.) and diabetes-related complications (such as dyslipidemia, hypertension, etc.), especially important confounding factors such as walking difficulties, depression, and social and economic status that are often overlooked^9^. Moreover, it is more important that elderly ophthalmic comorbidities related to DR, such as cataracts, glaucoma, and age-related macular degeneration, which seriously affect patient quality of life, are often not considered. Previous studies were mostly conducted in small patient populations (usually with a sample size of less than 2000), and still lacked grading evaluation of DR (only binary, i.e., with or without DR), trend changes and risk assessment of overall and specific mortality rates in DM patients. In addition, since randomization cannot be achieved in observational studies, previous models such as the COX regression model cannot fully adjust for the impact of important confounding factors between groups. Moreover, most previous studies may have overestimated the absolute risk of specific cause of death (such as CVD and DM) because they did not consider the risk of competing death^10^. Given the increasing concern about the prognosis of individuals with diabetes with an increasing risk of diabetic retinopathy (DR) and the attention paid to DR treatment, some studies have shown that the main treatment methods for diabetic retinopathy, such as intravitreal injection of anti-vascular endothelial growth factor, may lead to an increased risk of vascular thrombosis and thromboembolic events^11–13^. Therefore, it is necessary to clarify the exact impact of DR status and severity on the future all-cause and cause-specific mortality risks of individuals with diabetes, especially for cardiovascular disease and diabetes. This will help to understand whether DR can serve as a simple and effective predictive indicator recommended for early screening and risk stratification of individuals with diabetes.

The National Health and Nutrition Examination Survey (NHANES) is a study based on a nationally representative sample of non-institutionalized population in the United States, aimed at assessing the health and nutritional status of American adults and children. This program provides an opportunity to examine the relationship between DR and all-cause as well as cause-specific mortality, in the context of comprehensive population demographics, health-related behaviors, and comorbidities. To our knowledge, there are still no comprehensive reports on the use of appropriate models to analyze the relationship between specific causes of death and DR, not to mention analyzing differences in severity and trends in DR and adjusting for important confounders. Emily Frith et al. found that patients with mild and moderate/severe retinopathy had an increased risk of all-cause mortality (subdistribution hazard ratio (sHR) 1.81 and 4.14, respectively), compared to those without retinopathy^14^. Zhu et al. showed that the presence of retinopathy was associated with higher all-cause mortality (sHR 1.41; 95% CI 1.08–1.83)^8^. Given that DR is the most common form of retinal vascular disease, clarifying the relationship between specific cause of death and DR, and elucidating whether patients with specific comorbidities can benefit from retinal examination and DR grading status, has urgent and important clinical implications.

## Methods

### Retinal examination and retinopathy grading

The dataset from two NHANES cycles (2005–2006 and 2007–2008) was used for this study. Retinal imaging was performed for all non-institutionalized US civilians aged 40 years and older in a nearly completely dark room, obtaining two 45-degree non-mydriatic digital retinal images of each eye. Over 40 years of age are considered older persons. At least two experienced graders from the University of Wisconsin Ocular Epidemiology Reading Center, Madison, assessed the digital images of the fundus. The severity of retinal lesions was evaluated according to The Early Treatment Diabetic Retinopathy Study (ETDRS) grading scale^15^. In individuals with diabetes, eyes with worse retinal lesions were further classified into stages 10–80, and the severity of DR was divided into four levels (No DR, Mild NPDR, Moderate/severe NPDR, PDR) based on the retinopathy level. For detailed grading criteria, please refer to the NHANES Ophthalmology chapter in the NHANES Eye Examination Procedures Manual (Retinal Imaging (OPXRET_E)-OPDDRL4). The publicly available data used in this project were derived from the NHANES program, which was approved by the Ethics Review Board of the National Center for Health Statistics (NCHS), and in which all participants provided written informed consent to participate in the survey and agreed to the use of their data for health-related statistical research with links to vital statistics (e.g., the National Death Index), adhering to the principles of the Declaration of Helsinki.

### Mortality data

Using a probability matching algorithm, we determined the mortality rate data by matching the mortality rate archives of public access links in 2019 with the National Death Index (NDI) file^16^. The cause of death was determined according to the International Statistical Classification of Diseases and Related Health Problems, 10th Revision (ICD-10). Specifically, codes C00-C97 were determined as cancer-causes death, codes E10-E14 were determined as DM-causes death, code IG30 was determined as Alzheimer's disease (AD)-causes death, codes I00-I09, I11, I13, I20-I51 (heart disease) and I60-I69 (cerebrovascular disease) were determined as CVD-causes death, and the rest were considered as other-causes death. The follow-up time was calculated from the baseline interview date to the date of death or the end of the study review (December 31, 2019), whichever came first.

### Assessment of participant characteristics and covariates

Through interviews or physical examinations (including physiological measurements, laboratory tests, etc.), the following information was obtained: age (40–49, 50–59, 60–69, 70–79, and 80 years or older), sex (male and female), race/ethnicity (Non-Hispanic White, Non-Hispanic Black, Mexican American, or other), educational level (high school diploma obtained/not obtained), marital status (unmarried or other or married or living with a partner), poverty income ratio (PIR) (below poverty line (< 1.00) or at or above poverty line (≥ 1.00)), smoking status (never, former, or current), alcohol consumption (never (lifelong abstinence), former (ever drank alcohol), current heavy use (≥ 3 drinks per day for females or ≥ 4 drinks per day for males or binge drinking on 5 or more days per month), current moderate use (≥ 2 drinks per day for females or ≥ 3 drinks per day for males or binge drinking ≥ 2 days per month) and current mild use (not meeting the above criteria)), body mass index (BMI) (defined as weight in kilograms divided by height in meters squared, normal to overweight (18.5–30.0), underweight (< 18.5), or obese (≥ 30.0)), diabetes (doctor told you have diabetes, or glycohemoglobin HbA1c (%) > 6.5, or fasting glucose (mmol/l) ≥ 7.0, or random blood glucose (mmol/l) ≥ 11.1, or two-hour OGTT blood glucose (mmol/l) ≥ 11.1, or use of diabetes medication or insulin), hypertension (self-reported history of hypertension, use of antihypertensive medication, or systolic blood pressure of 140 mm Hg or higher and/or diastolic blood pressure of 90 mm Hg or higher based on the lowest of 3 measurements), hyperlipidemia or dyslipidemia (high triglycerides (TG) (TG ≥ 150 mg/dl), and/or high total cholesterol (TC) (TC ≥ 200 mg/dl), and/or low-density lipoprotein cholesterol (LDL-C) ≥ 130 mg/dl, and/or high-density lipoprotein cholesterol (HDL) < 40 mg/dl (male), 50 mg/dl (female) (converted to mmol/L by multiplying by 0.0259) or use of lipid-lowering drugs), high C-reactive protein level (CRP) (CRP content of at least 1 mg/dl), depressive symptoms/9-item Patient Health Questionnaire (PHQ-9) score (a score of 10 or higher is considered to have depressive symptoms)^17^, walking disability (self-reported questionnaire response or the need for special equipment to assist walking), self-rated health status (poor to fair and good to excellent), systemic comorbidity (including doctor-diagnosed congestive heart failure, coronary heart disease, angina, heart attack, stroke, and cancer), age-related ophthalmic comorbidity (including cataracts, AMD, and glaucoma) based on questionnaire surveys and/or retinal imaging, with specific diagnostic criteria consistent with previous studies^18,19^.

### Statistical analysis

The data sets from the two NHANES cycles (2005–2006 and 2007–2008) were analyzed using the complex stratified design analysis method provided by NHANES analysis and reporting guidelines. Continuous variables and categorical variables were described using mean (SE) and numbers (weighted percentages), respectively, to describe the baseline characteristics of all participants and the matched baseline characteristics after propensity score matching (PSM). Unpaired t-tests and Rao-Scott Pearson χ2 tests were used to compare the distribution of design-adjusted continuous or categorical variable data and mortality characteristics. Multiple adjusted Kaplan–Meier estimation was used to generate survival curves for patients with DR. Subdistribution hazard ratios (sHRs) and 95% confidence intervals (CIs) were estimated using Cox proportional hazards regression models adjusted for age and sex. The final Cox proportional hazards regression model included covariates that were significantly associated with mortality and DR. To screen for covariates, we performed the directed acyclic graph of DR and mortality risk based on previously published studies^20–28^. Interaction test results confirmed that there was no statistically significant interaction between DR and each covariate (*P* > 0.05). By calculating the interaction between each covariate and follow-up time, it was confirmed that each covariate satisfied the proportional hazards assumption (PH) (*P* > 0.05). Multifactor-adjusted Fine and Gray competing risk regression models estimated the risk of specific causes of death to address the bias caused by other causes of death being considered as competing risks for a specific cause of death^18,29^.

In order to address the non-linear relationship between age and mortality, we adjusted for age and age squared in the Cox proportional hazards regression model or Fine and Gray competing risks regression model for sensitivity analysis^18^. The variance inflation factor (VIF) was used to test for collinearity effects among all covariates. The PSM method was used to address the issue of imbalanced covariates between groups in observational studies, achieving effects like those of randomized controlled trials and enabling more reasonable comparisons between different observation groups^30–32^. We used the “MatchIt” package in the R software to perform 1:1 propensity score matching for DR and non-DR patients, and the baseline data differences between the two groups after PSM were not statistically significant, indicating that the two groups were relatively balanced and comparable (Table S1), with all baseline characteristics of participants used as matching variables. We used various packages in R version 4.2.1 (2022-06-23), including “nhanesR,” “survey,” “reshape2,” “do,” and “dplyr,” for all statistical analyses, and P-values less than 0.05 were considered to have statistically significant differences.

## Results

This study initially covered 6797 participants aged 40 years or older. Among them, 1093 participants (16.08%) without gradable retinal images and 4382 participants (64.47%) without diabetes diagnosis were excluded. Participants with missing values of baseline characteristics were then excluded. After screening, a total of 1077 participants (15.85%) were included in the final analysis (Figure S1 in the supplementary material). Compared to the excluded participants, the included participants were younger (≥ 80 years, 81 (6.06%) vs 38 (14.92%); *P* < 0.0001), more likely to be white (484 (70.73%) vs 79 (53.82%); *P* = 0.001), had higher baseline income (868 (88.30%) vs 100 (77.10%); *P* = 0.001), difficulty walking (900 (84.21%) vs 173 (71.60%)), and history of congestive heart failure, angina, heart attack, stroke (Table S2 in the supplementary material).

Table 1 presents the baseline characteristics of the participants, including demographic and systemic disease history, stratified by the presence and severity of DR. The results showed significant differences in gender, education level, walking difficulty, history of congestive heart failure, stroke, and comorbid ocular diseases between DR and non-DR patients. Similar results were observed when stratifying by DR severity, where the trend test suggested statistically significant differences in age, gender, race, high C-reactive protein level, walking difficulty, history of congestive heart failure, angina, and comorbid ocular diseases. In this study, the VIF for all covariates was less than 2 (mean [SE], 1.26 [0.07]). Figure S2 showed the directed acyclic graph of the included covariates relating to DR and mortality risk.

**Table 1:** Demographic, health behavior, and general health characteristics of participants by diabetic retinopathy status^a^.

Characteristic	Study participants							
	All (n = 1077) N = 3,025,316	No diabetic retinopathy (n = 773) N = 2,277,780	Diabetic retinopathy (n = 304) N = 747,535.8	*P*^b^	Mild NPDR (n = 214) N = 547,914.5	Moderate to severe NPDR (n = 69) N = 163,008.2	PDR (n = 21) N = 36,613.09	*P* for trend
Age, No. (%), y				0.28				0.01
40–49	136 (12.63)	101 (19.21)	35 (16.44)		23 (16.12)	11 (19.87)	1 (5.99)	
50–59	255 (23.68)	191 (31.00)	64 (26.16)		45 (24.52)	16 (35.36)	3 (9.79)	
60–69	365 (33.89)	257 (27.30)	108 (27.16)		68 (25.43)	24 (20.85)	16 (81.06)	
70–79	240 (22.28)	168 (17.15)	72 (21.98)		57 (24.61)	14 (17.35)	1 (3.17)	
≥ 80	81 (7.52)	56 (5.34)	25 (8.26)		21 (9.32)	4 (6.56)	0 (0.00)	
Sex, No. (%)				0.003				0.03
Male	559 (51.9)	387 (48.43)	172 (57.88)		123 (58.54)	39 (53.21)	10 (68.75)	
Female	518 (48.1)	386 (51.57)	132 (42.12)		91 (41.46)	30 (46.79)	11 (31.25)	
Race/ethnicity, No. (%)				0.05				0.02
Non-Hispanic white	484 (44.94)	365 (71.95)	119 (67.01)		94 (69.49)	22 (62.28)	3 (51.05)	
Non-Hispanic black	283 (26.28)	186 (12.59)	97 (19.23)		60 (16.94)	28 (24.09)	9 (31.80)	
Mexican American	200 (18.57)	140 (6.96)	60 (8.05)		40 (6.93)	16 (11.96)	4 (7.34)	
Other	110 (10.21)	82 (8.50)	28 (5.71)		20 (6.63)	3 (1.67)	5 (9.81)	
Marital status, No. (%)				0.53				0.6
Unmarried or other	413 (38.35)	293 (32.13)	120 (34.71)		81 (33.58)	27 (35.46)	12 (48.15)	
Married or living with a partner	664 (61.65)	480 (67.87)	184 (65.29)		133 (66.42)	42 (64.54)	9 (51.85)	
Educational attainment, No. (%)				0.03				0.06
< High school	414 (38.44)	279 (22.99)	135 (31.43)		91 (29.95)	31 (30.35)	13 (58.23)	
≥ High school	663 (61.56)	494 (77.01)	169 (68.57)		123 (70.05)	38 (69.65)	8 (41.77)	
Poverty income ratio, No. (%)				0.61				0.48
Below poverty line (< 1.00)	209 (19.41)	152 (11.46)	57 (12.43)		36 (10.90)	15 (17.03)	6 (14.87)	
At or above poverty line (≥ 1.00)	868 (80.59)	621 (88.54)	247 (87.57)		178 (89.10)	54 (82.97)	15 (85.13)	
Alcohol consumption, No. (%)				0.07				0.22
Never	185 (17.18)	128 (15.79)	57 (19.55)		37 (19.17)	17 (23.08)	3 (9.49)	
Former	382 (35.47)	260 (30.60)	122 (36.40)		84 (33.86)	26 (39.90)	12 (58.83)	
Mild	306 (28.41)	227 (30.97)	79 (31.49)		60 (33.45)	16 (26.58)	3 (24.03)	
Moderate	109 (10.12)	85 (14.57)	24 (6.53)		18 (7.51)	4 (3.67)	2 (4.64)	
Heavy	95 (8.82)	73 (8.07)	22 (6.03)		15 (6.01)	6 (6.78)	1 (3.01)	
BMI, No. (%)				0.67				0.73
18.5–30.0	465 (43.18)	320 (38.16)	145 (40.51)		107 (43.02)	31 (35.03)	7 (27.41)	
< 18.5	5 (0.46)	4 (0.29)	1 (0.13)		1 (0.18)	0 (0.00)	0 (0.00)	
≥ 30.0	607 (56.36)	449 (61.54)	158 (59.36)		106 (56.80)	38 (64.97)	14 (72.59)	
High C-reactive protein level, No. (%)				0.08				0.01
No	884 (82.08)	625 (79.07)	259 (85.05)		189 (88.60)	57 (81.75)	13 (46.64)	
Yes	193 (17.92)	148 (20.93)	45 (14.95)		25 (11.40)	12 (18.25)	8 (53.36)	
Hypertension, No. (%)				0.91				0.31
No	279 (25.91)	214 (28.19)	65 (27.74)		49 (29.21)	14 (28.49)	2 (2.43)	
Yes	798 (74.09)	559 (71.81)	239 (72.26)		165 (70.79)	55 (71.51)	19 (97.57)	
Hyperlipidemia, No. (%)				0.92				0.76
No	128 (11.88)	92 (11.18)	36 (11.50)		26 (11.25)	8 (13.80)	2 (4.91)	
Yes	949 (88.12)	681 (88.82)	268 (88.50)		188 (88.75)	61 (86.20)	19 (95.09)	
Depressive symptoms, No. (%)				0.38				0.05
No	963 (89.42)	683 (89.27)	280 (91.86)		199 (94.49)	61 (81.81)	20 (97.17)	
Yes	114 (10.58)	90 (10.73)	24 (8.14)		15 (5.51)	8 (18.19)	1 (2.83)	
Difficulty walking, No. (%)				0.02				0.03
No	900 (83.57)	655 (85.60)	245 (79.96)		177 (81.63)	55 (78.30)	13 (62.32)	
Yes	177 (16.43)	118 (14.40)	59 (20.04)		37 (18.37)	14 (21.70)	8 (37.68)	
Health status, No. (%)				0.15				0.35
Poor to fair	463 (42.99)	315 (34.00)	148 (39.34)		94 (36.61)	41 (46.96)	13 (46.30)	
Good to excellent	614 (57.01)	458 (66.00)	156 (60.66)		120 (63.39)	28 (53.04)	8 (53.70)	
History of congestive heart failure, No. (%)				< 0.001				< 0.001
No	982 (91.18)	725 (94.30)	257 (85.11)		184 (85.56)	60 (88.56)	13 (63.06)	
Yes	95 (8.82)	48 (5.70)	47 (14.89)		30 (14.44)	9 (11.44)	8 (36.94)	
History of coronary heart disease, No. (%)				0.28				0.34
No	963 (89.42)	698 (89.46)	265 (86.02)		187 (84.93)	63 (90.45)	15 (82.60)	
Yes	114 (10.58)	75 (10.54)	39 (13.98)		27 (15.07)	6 (9.55)	6 (17.40)	
History of angina, No. (%)				0.24				0.04
No	1006 (93.41)	727 (93.85)	279 (91.58)		194 (89.49)	68 (98.59)	17 (91.69)	
Yes	71 (6.59)	46 (6.15)	25 (8.42)		20 (10.51)	1 (1.41)	4 (8.31)	
History of heart attack, No. (%)				0.28				0.28
No	962 (89.32)	701 (90.31)	261 (87.67)		183 (86.21)	62 (91.96)	16 (90.53)	
Yes	115 (10.68)	72 (9.69)	43 (12.33)		31 (13.79)	7 (8.04)	5 (9.47)	
History of stroke, No. (%)				0.03				0.15
No	975 (90.53)	711 (92.11)	264 (86.74)		189 (87.17)	59 (87.59)	16 (76.59)	
Yes	102 (9.47)	62 (7.89)	40 (13.26)		25 (12.83)	10 (12.41)	5 (23.41)	
History of cancer, No. (%)				0.5				0.32
No	927 (86.07)	664 (85.23)	263 (87.13)		181 (84.98)	64 (93.39)	18 (91.34)	
Yes	150 (13.93)	109 (14.77)	41 (12.87)		33 (15.02)	5 (6.61)	3 (8.66)	
History of comorbid ocular diseases, No. (%)				0.002				< 0.001
No	718 (66.67)	538 (72.79)	180 (60.95)		133 (63.88)	41 (60.12)	6 (20.87)	
Yes	359 (33.33)	235 (27.21)	124 (39.05)		81 (36.12)	28 (39.88)	15 (79.13)	

*NPDR* Non-proliferative diabetic retinopathy, *PDR* Proliferative diabetic retinopathy; *BMI* Body mass index (calculated as weight in kilograms divided by height in meters squared).

^a^All proportions, means, and SEs are weighted estimates of the US population characteristics, taking into account the complex sampling design of the National Health and Nutrition Examination Survey.

^b^All *P* values were calculated using the unpaired t test for continuous variables and the design-adjusted Rao-Scott Pearson χ^2^ test for categorical variables. Comparisons were between each group with retinopathy and the group with no retinopathy and were unadjusted.

### All-cause mortality

According to the results shown in Table 2, among the 1077 participants included in the study, 304 individuals (28.23%) had DR, with 214 individuals (19.87%) having mild NPDR, 69 individuals (6.41%) having moderate to severe NPDR, and 21 individuals (1.95%) having PDR. As of December 31, 2020, at a median follow-up of 12.24 years (interquartile range, 11.16–13.49 years), 379 participants were identified as deceased, with 43.90% of those had DR at baseline, including 41.50% with mild NPDR, 46.77% with moderate to severe NPDR, and particularly high mortality of 67.21% in those with PDR, significantly higher than those without DR (26.85%). The mean (SE) age at death was significantly younger in non-DR participants (60.01 (0.58) years) compared with those with DR (62.27 (0.78) years), mild NPDR (62.85 (0.83) years), and PDR (63.04 (1.41) years) (Table 2). In addition, the survival time was significantly longer in non-DR participants (135.58 (1.92) months) compared with DR participants (122.35 (3.60) months), those with mild NPDR (123.87 (4.16) months), those with moderate to severe NPDR (123.62 (6.12) months), and those with PDR (93.97 (27.59) months) (Table 2).

**Table 2:** Mortality characteristics overall and by different diabetic retinopathy status^a^.

Characteristics	Diabetic retinopathy status^b^						
	All (n = 1077) N = 3,025,316	No diabetic retinopathy (n = 773) N = 2,277,780	Diabetic retinopathy (n = 304) N = 747,535.8	Mild NPDR (n = 214) N = 547,914.5	Moderate to severe NPDR (n = 69) N = 163,008.2	PDR (n = 21) N = 36,613.09	*P* for trend
Age at death, mean (SE), y							
Due to all causes	60.57 (0.47)	60.01 (0.58)	62.27 (0.78)*	62.85 (0.83)*	60.15 (1.69)	63.04 (1.41)*	**0.02**
Due to cancer	67.52 (1.18)	67.80 (1.52)	66.58 (2.32)	69.98 (2.27)	62.16 (2.62)	58.83 (1.72)*	**0.01**
Due to CVD	69.27 (0.78)	69.52 (1.10)	68.90 (1.34)	69.82 (1.44)*	68.12 (3.64)	58.01 (4.72)	**0.03**
Due to DM	64.26 (2.78)	60.60 (3.91)	67.89 (2.59)	71.56 (3.42)	64.25 (2.65)	60.27 (0.00)**	**0.001**
Due to other causes	68.84 (1.38)	69.84 (1.72)	66.71 (2.36)	67.48 (3.14)	64.25 (5.28)	66.22 (0.78)****	** < 0.0001**
Mortality rate, No. (%)							
Due to all causes	379 (35.19)	243 (26.85)	136 (43.90)***	89 (41.50)***	31 (46.77)*	16 (67.21)*	**0.001**
Due to cancer	77 (7.15)	57 (5.90)	20 (5.37)	12 (4.37)	5 (8.20)	3 (7.81)	0.53
Due to CVD	140 (13)	85 (9.37)	55 (18.65)****	37 (18.98)***	12 (17.63)*	6 (18.28)*	** < 0.0001**
Due to DM	35 (3.25)	17 (2.03)	18 (6.24)****	12 (4.91)*	4 (7.99)*	2 (18.44)**	**0.001**
Due to other causes	127 (11.79)	84 (9.55)	43 (13.64)*	28 (13.23)	10 (12.96)	5 (22.68)	0.15
Time to death from baseline examination, mean (SE), mo							
Due to all causes	132.31 (1.65)	135.58 (1.92)	122.35 (3.60)*	123.87 (4.16)*	123.62 (6.12)	93.97 (27.59)	**0.03**
Due to cancer	71.95 (6.33)	70.15 (6.78)	77.96 (11.85)	86.00 (14.03)	63.64 (14.87)	77.58 (18.36)	0.89
Due to CVD	83.91 (5.53)	84.48 (8.19)	83.04 (4.80)	83.34 (5.34)*	86.00 (7.35)	65.59 (16.68)	**0.04**
Due to DM	92.42 (11.04)	88.72 (10.64)	96.08 (17.39)	86.44 (22.82)	113.66 (17.53)	100.60 (0.00)	0.25
Due to other causes	88.41 (6.25)	94.78 (5.95)	74.80 (10.83)	81.21 (13.58)	70.11 (8.58)	30.75 (21.37)	0.22

*NPDR* Non-proliferative diabetic retinopathy, *PDR* Proliferative diabetic retinopathy, *CVD* Cardiovascular disease, *DM* Diabetes mellitus.

^a^Mortality was assessed through December 31, 2020. All proportions, means, and SEs are weighted estimates of the US population characteristics, taking into account the complex sampling design of the National Health and Nutrition Examination Survey.

^b^All *P* values were calculated using the unpaired t test for continuous variables and the design-adjusted Rao–Scott Pearson χ^2^ test for categorical variables. Comparisons were between each group with retinopathy and the group with no retinopathy and were unadjusted.

*P < 0.05; **P < 0.01; ***P < 0.001; ****P < 0.0001.

Significant values are in bold.

We used the Cox proportional hazards regression model, adjusted for age and sex, to analyze the correlation between various covariates at baseline and all-cause mortality (Table 3). Stratified analysis by age was performed for participants aged 40 years and older, and the sHRs increased exponentially with every 10-year increase in age. Women (sHR = 0.59; 95% CI 0.47–0.74) and Mexican Americans (sHR = 0.71; 95% CI 0.50–1.01) had a significantly lower risk of all-cause mortality. In addition, educational attainment above a high school diploma (sHR = 0.65; 95% CI 0.53–0.80), being married or living with a partner (sHR = 0.57; 95% CI 0.46–0.71), being at or above the poverty line (sHR = 0.49; 95% CI 0.36–0.66), and reporting good to excellent self-rated health (sHR = 0.48; 95% CI 0.38–0.61) were associated with a lower risk of all-cause mortality. Symptoms of depression (sHR = 1.84; 95% CI 1.42–2.40), walking difficulties (sHR = 2.98; 95% CI 2.12–4.19), and self-reported history of CVD (e.g., history of congestive heart failure, sHR = 2.84; 95% CI 2.14–3.78) were significantly associated with a higher risk of all-cause mortality.

**Table 3:** Due to all causes mortality by demographic, health-related behaviors and general health characteristics^a^.

Characteristics	Participants		
	Survived (n = 698) N = 2,085,487	Died (n = 379) N = 939,829.7	sHR (95% CI)^b^
Age, No. (%), y			
40–49	116 (23.47)	20 (7.55)	1 [Reference]
50–59	211 (37.09)	44 (13.64)	1.15 (0.58–2.31)
60–69	254 (27.56)	111 (26.61)	2.67 (1.53–4.66)***
70–79	107 (10.69)	133 (35.33)	6.56 (3.80–11.34)****
≥ 80	10 (1.18)	71 (16.88)	18.83 (10.29–34.44)****
Sex, No. (%)			
Male	337 (48.04)	222 (56.81)	1 [Reference]
Female	361 (51.96)	157 (43.19)	0.59 (0.47–0.74)****
Race/ethnicity, No. (%)			
Non-Hispanic white	261 (67.38)	223 (78.16)	1 [Reference]
Non-Hispanic black	195 (15.05)	88 (12.40)	0.98 (0.77–1.26)
Mexican American	160 (8.50)	40 (4.42)	0.71 (0.50–1.01)
Other	82 (9.07)	28 (5.01)	0.96 (0.47–1.96)
Marital status, No. (%)			
Unmarried or other	231 (27.10)	182 (45.34)	1 [Reference]
Married or living with a partner	467 (72.90)	197 (54.66)	0.57 (0.46–0.71)****
Educational attainment, No. (%)			
< High school	252 (20.35)	162 (35.55)	1 [Reference]
≥ High school	446 (79.65)	217 (64.45)	0.65 (0.53–0.80)****
Poverty income ratio, No. (%)			
Below poverty line (< 1.00)	127 (9.40)	82 (16.80)	1 [Reference]
At or above poverty line (≥ 1.00)	571 (90.60)	297 (83.20)	0.49 (0.36–0.66)****
Alcohol consumption, No. (%)			
Never	115 (15.56)	70 (19.29)	1 [Reference]
Former	223 (28.07)	159 (40.82)	1.08 (0.70–1.66)
Mild	207 (32.58)	99 (27.80)	0.67 (0.44–1.01)
Moderate	82 (14.64)	27 (8.03)	0.76 (0.38–1.52)
Heavy	71 (9.14)	24 (4.07)	0.64 (0.30–1.38)
BMI, No. (%)			
18.5–30.0	273 (34.93)	192 (47.20)	1 [Reference]
< 18.5	2 (0.22)	3 (0.32)	1.40 (0.66–2.96)
≥ 30.0	423 (64.84)	184 (52.48)	0.99 (0.81–1.20)
High C-reactive protein level, No. (%)			
No	573 (80.19)	311 (81.36)	1 [Reference]
Yes	125 (19.81)	68 (18.64)	1.31 (0.91–1.88)
Hypertension, No. (%)			
No	200 (31.52)	79 (20.44)	1 [Reference]
Yes	498 (68.48)	300 (79.56)	1.12 (0.84–1.50)
Hyperlipidemia, No. (%)			
No	77 (10.50)	51 (12.94)	1 [Reference]
Yes	621 (89.50)	328 (87.06)	0.74 (0.52–1.04)
Depressive symptoms, No. (%)			
No	635 (91.26)	328 (86.91)	1 [Reference]
Yes	63 (8.74)	51 (13.09)	1.84 (1.42–2.40)****
Difficulty walking, No. (%)			
No	626 (91.39)	274 (68.27)	1 [Reference]
Yes	72 (8.61)	105 (31.73)	2.98 (2.12–4.19)****
Health status, No. (%)			
Poor to fair	279 (30.51)	184 (45.97)	1 [Reference]
Good to excellent	419 (69.49)	195 (54.03)	0.48 (0.38–0.61)****
History of congestive heart failure, No. (%)			
No	668 (96.42)	314 (82.30)	1 [Reference]
Yes	30 (3.58)	65 (17.70)	2.84 (2.14–3.78)****
History of coronary heart disease, No. (%)			
No	653 (92.33)	310 (80.35)	1 [Reference]
Yes	45 (7.67)	69 (19.65)	1.48 (1.11–1.97)**
History of angina, No. (%)			
No	667 (95.61)	339 (88.15)	1 [Reference]
Yes	31 (4.39)	40 (11.85)	1.49 (0.95–2.34)
History of heart attack, No. (%)			
No	656 (94.02)	306 (79.97)	1 [Reference]
Yes	42 (5.98)	73 (20.03)	1.77 (1.35–2.31)****
History of stroke, No. (%)			
No	655 (94.54)	320 (82.44)	1 [Reference]
Yes	43 (5.46)	59 (17.56)	1.78 (1.23–2.58)**
History of cancer, No. (%)			
No	627 (88.45)	300 (79.58)	1 [Reference]
Yes	71 (11.55)	79 (20.42)	1.14 (0.79–1.66)
History of comorbid ocular diseases, No. (%)			
No	531 (78.74)	187 (50.17)	1 [Reference]
Yes	167 (21.26)	192 (49.83)	1.27 (0.96–1.68)

*NPDR* Non-proliferative diabetic retinopathy, *PDR* Proliferative diabetic retinopathy; *BMI* Body mass index (calculated as weight in kilograms divided by height in meters squared); *sHR* Subdistribution hazard ratio.

^a^All-cause mortality was assessed through December 31, 2020. All proportions, means, and SEs are weighted estimates of the US population characteristics, taking into account the complex sampling design of the National Health and Nutrition Examination Survey.

^b^Adjusted for age and sex.

*P < 0.05; **P < 0.01; ***P < 0.001; ****P < 0.0001.

After adjusting for confounding factors significantly associated with both mortality and DR, we performed multivariate regression analysis using Cox Proportional Hazards Models. The results showed that compared with participants without DR at baseline, any DR (sHR = 1.53), Mild NPDR (sHR = 1.38), and moderate to severe NPDR (sHR = 1.96) were significantly associated with all-cause mortality (Table 4). However, there was no statistically significant difference for PDR, but the trend test suggested a significant difference in the increase of all-cause mortality risk with the severity of DR (*P* = 0.01) (Table 4).

**Table 4:** Cox proportional hazards models for all-cause mortality and fine and gray competing risks regression models for specific-cause mortality by diabetic retinopathy status.

Retinopathy status	Mortality														
	Due to all causes n = 379 N = 939,829.7			Due to cancer n = 77 N = 174,580.2			Due to CVD n = 140 N = 352,857.5			Due to DM n = 35 N = 92,925.73			Due to other causes n = 127 N = 319,466.3		
	sHR^a^ (95% CI)	sHR^b^ (95% CI)	sHR^c^ (95% CI)	sHR^a^ (95% CI)	sHR^b^ (95% CI)	sHR^c^ (95% CI)	sHR^a^ (95% CI)	sHR^b^ (95% CI)	sHR^c^ (95% CI)	sHR^a^ (95% CI)	sHR^b^ (95% CI)	sHR^c^ (95% CI)	sHR^a^ (95% CI)	sHR^b^ (95% CI)	sHR^c^ (95% CI)
No Diabetic Retinopathy (n = 773) N = 2,277,780	1 [Reference]	1 [Reference]	1 [Reference]	1 [Reference]	1 [Reference]	1 [Reference]	1 [Reference]	1 [Reference]	1 [Reference]	1 [Reference]	1 [Reference]	1 [Reference]	1 [Reference]	1 [Reference]	1 [Reference]
Any Diabetic Retinopathy (n = 304) N = 747,535.8	1.84 (1.40–2.43)****	1.53 (1.14–2.06)**	1.52 (1.12–2.05)**	1.17 (0.59–2.34)	0.95 (0.45–2.00)	0.95 (0.45–2.01)	2.36 (1.60–3.48)****	1.89 (1.31–2.73)***	1.87 (1.29–2.71)***	3.82 (1.82–8.04)***	3.51 (1.74–7.06)***	3.63 (1.83–7.23)***	1.81 (1.11–2.95)*	1.46 (0.82–2.59)	1.36 (0.75–2.47)
Mild NPDR (n = 214) N = 547,914.5	1.72 (1.29–2.30)***	1.38 (1.00–1.91)*	1.37 (0.99–1.91)	0.92 (0.40–2.14)	0.70 (0.32–1.54)	0.70 (0.32–1.55)	2.33 (1.57–3.45)****	1.82 (1.21–2.71)**	1.83 (1.21–2.75)**	2.98 (1.37–6.51)**	2.65 (1.27–5.50)**	2.68 (1.28–5.60)**	1.71 (0.92–3.17)*	1.31 (0.64–2.69)	1.20 (0.56–2.59)
Moderate to Severe NPDR (n = 69) N = 163,008.2	1.94 (1.25–3.00)**	1.96 (1.23–3.11)**	1.83 (1.16–2.87)**	1.84 (0.50–6.77)	2.39 (0.52–11.00)	2.37 (0.53–10.61)	2.25 (1.21–4.21)**	2.12 (1.27–3.53)**	1.91 (1.12–3.25)*	4.75 (1.53–14.76)**	5.87 (1.60–21.59)**	6.70 (1.97–22.76)**	1.75 (0.87–3.49)	2.14 (1.27–3.62)*	1.85 (1.17–2.94)*
PDR (n = 21) N = 36,613.09	3.80 (1.06–13.61)*	2.60 (0.47–14.40)	2.99 (0.52–17.14)	2.71 (0.53–13.81)	1.08 (0.11–10.50)	1.17 (0.12–11.96)	3.82 (1.01–14.51)*	2.35 (0.43–12.86)	2.63 (0.47–14.71)	14.68 (2.12–101.53)**	8.86 (1.12–69.87)*	13.56 (1.49–123.16)*	5.12 (0.43–60.56)	1.93 (0.10–37.86)	2.64 (0.13–53.46)
*P* for trend	< 0.0001	0.01	0.01	0.38	0.72	0.74	< 0.0001	0.004	0.004	< 0.0001	< 0.0001	< 0.0001	0.01	0.16	0.18

*NPR* Non-proliferative retinopathy; *PR* Proliferative retinopathy; *CVD* Cardiovascular disease; *DM* Diabetes mellitus; *sHR* Subdistribution hazard ratio.

^a^Unadjusted for confounding factors.

^b^Adjusted for age, sex, race/ethnicity, educational attainment, marital status, body mass index, family income, smoking status, alcohol consumption, hypertension, high C-reactive protein level, depressive symptoms, walking disability, self-rated health, history of coronary heart disease, congestive heart failure, heart attack, stroke, angina and comorbid ocular diseases.

^c^Adjusted for age, squared age, sex, race/ethnicity, educational attainment, marital status, body mass index, family income, smoking status, alcohol consumption, hypertension, high C-reactive protein level, depressive symptoms, walking disability, self-rated health, history of coronary heart disease, congestive heart failure, heart attack, stroke, angina and comorbid ocular diseases.

*P < 0.05; **P < 0.01; ***P < 0.001; ****P < 0.0001.

**Figure 1:**
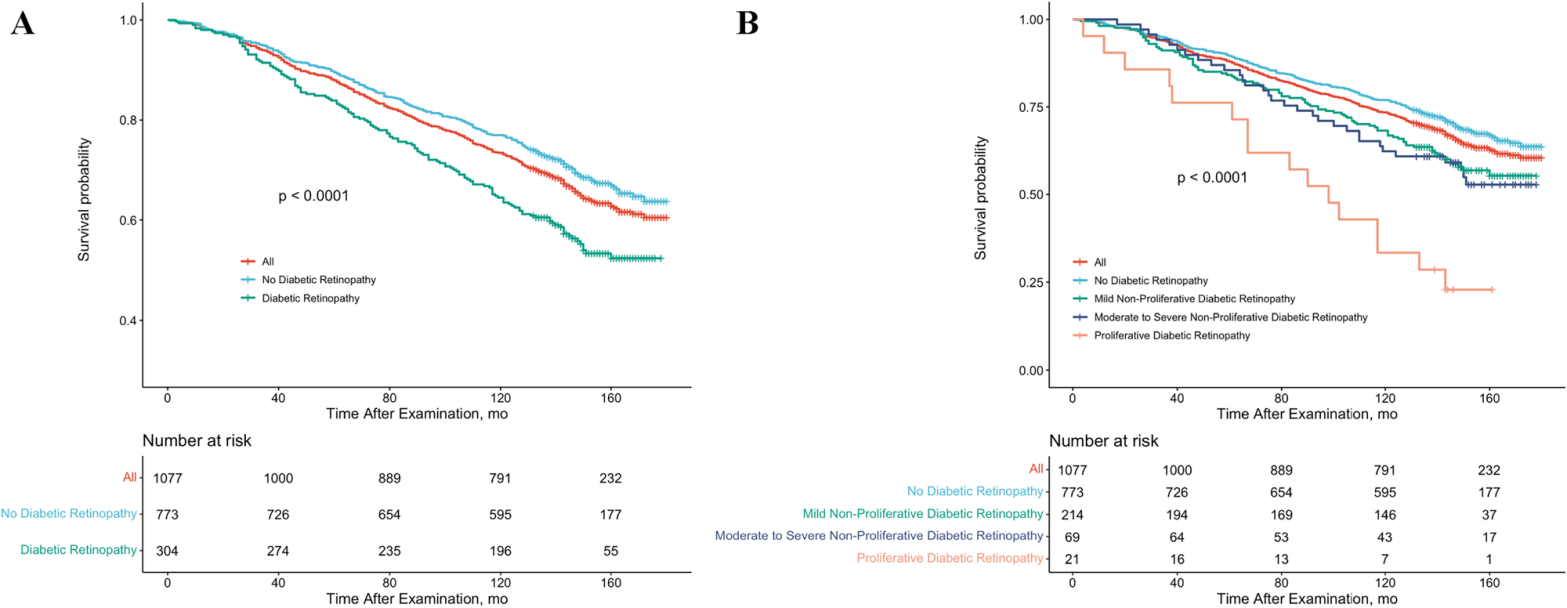
Multiple adjusted Kaplan–Meier Curve for All-Cause Mortality Rate by Diabetic Retinopathy (DR) Status. Study results were stratified according to DR status (**A**) presence or absence of DR (**B**) DR status grading, using 2005–2008 National Health and Nutrition Examination Survey data. All-cause mortality was assessed through 31 December 2020.

In addition, by using the PSM method to balance the covariates between the two groups of participants matched 1:1, a COX model was recreated that included 304 non-DR patients and 304 DR patients. Table S3 showed that a total of 242 participants died from all-causes, representing a non-hospitalized population of 210,503 residents in the United States. The results once again confirmed that, compared to non-DR patients, DR patients had increased risk of all-cause mortality (sHR = 1.35; 95% CI 1.03–1.78) (Table S3). Figure 1 shows the multiple adjusted Kaplan–Meier curves for all-cause mortality based on the presence and severity of DR. The results indicated that individuals with diabetes with DR had significantly lower survival probabilities during follow-up than those without retinopathy, and results grouped according to the severity of DR showed progressive decreases as the severity of DR increased.

### Cause-specific mortality

Among the 379 deceased participants in the study, 77 (20.32%) died of cancer, 140 (36.94%) died of CVD, 35 (9.23%) died of DM, and 127 (33.51%) died of other causes (Table 2). The results in Table 4 show that, after adjusting for covariates, any DR and mild to moderate or severe NPDR are associated with increased risks of death due to CVD and DM-specific causes compared to participants without DR, and only PDR is significantly associated with an increased DM-specific mortality. Compared to patients without DR, participants with any DR have a nearly twofold increased risk of death due to CVD (sHR = 1.89) and more than a threefold increased risk of death due to DM (sHR = 3.51). It is worth noting that the sHR for DM increases exponentially with the severity of DR, from 2.65 in mild NPDR to 8.86 in the PDR group. Trend tests show significant differences in the risk of death attributable to CVD and DM with increasing severity of DR (*P* for trend = 0.004 and < 0.0001, respectively) (Table 4).

The results after PSM also confirm that the increased risks of death due to CVD-specific (sHR = 1.71; 95% CI 1.07–2.73) and DM-specific (sHR = 5.56; 95% CI 2.28–13.59) causes among participants with DR compared to those without DR are statistically significant, with no significant difference observed in cancer-specific and other causes of death (Table S3).

### Sensitivity analyses

We included age squared in the Cox proportional hazards model (for all-cause mortality) and the Fine and Gray competing risks regression model (for specific-cause mortality) to adjust for the non-linear relationship between age and mortality. The results remained consistent with the previous results (Table 4).

## Discussion

Our study results suggest that the presence of any DR, as well as mild, moderate to severe NPDR, are significantly associated with an increased risk of all-cause mortality and CVD-specific mortality. The presence of any DR and its severity are also significantly associated with an increased risk of DM-specific mortality, and the risk of mortality due to DM increases exponentially with the severity of DR.

Several studies related to our research topic have been reported, such as the CHAMP1ON study^33^, the Catalonia study^34^, and the Swedish cohort study^35^, which support our research conclusion that the presence and severity of DR strongly affect the survival outcome of patients. However, it is worth noting that, in addition to any DR, mild to moderate NPDR, we did not find a significant association between the highest severity of DR, or PDR, and all-cause mortality. This is in contrast to the results reported in the RIACE cohort study^36^ and the SIDIAP cohort study (n = 22,402)^34^ targeting type 2 diabetes patients, which may be most likely attributable to their overestimation of mortality risk due to inadequate adjustment for confounding factors, in addition to differences in participants' ethnicity, type of diabetes and DR classification methods. An 18-year follow-up cohort study (n = 425) classified participants into no retinopathy, background retinopathy, and PDR based on standardized clinical fundus examination, and found that PDR was significantly associated with type 2 diabetes mortality after adjusting for traditional risk factors^37^. Different studies have varied in their evaluation and grading criteria for DR, leading to high heterogeneity in the data and difficulties in direct comparison. Another DR grading strategy by Barrot et al. was based on the Early Treatment Diabetic Retinopathy Study (ETDRS) classification into no apparent retinopathy (NDR), mild non-proliferative retinopathy (NPDR), moderate NPDR, severe NPDR, proliferative diabetic retinopathy (PDR), and diabetic macular edema (DME), and found that PDR was significantly higher than NPDR in terms of effect size on overall mortality risk^34^. However, The EURODIAB Prospective Complications Study compared color retinal photographs with standard photographs and classified them into NPDR and PDR. After multivariate analysis, it was found that there was no significant association between PDR and all-cause mortality in individuals with diabetes (OR = 2.06, 95% CI 0.63–6.73)^38^. Therefore, compared to other studies, our study adjusts for more adequate confounding and classifies DR according to the ETDRS grading scale, but further long-term studies with large samples are needed to confirm the relationship between all-cause mortality and PDR in the US population.

Similarly, our study confirms a significant correlation between the occurrence of DR and the CVD-caused mortality risk in US non-institutional patients, consistent with the pooling results of an umbrella review of meta-analyses^39^ that included 34 studies, suggesting a significant positive correlation between DR and increased CVD-caused mortality. Similar conclusions were also observed in a large Asian cohort study that included 10,033 participants^40^, indicating that the occurrence of DR in individuals with diabetes is independently and significantly associated with an increased CVD-caused mortality. The study by Modjtahedi et al. also demonstrated that the severity of DR is significantly correlated with the future occurrence of CVD, such as cerebrovascular disease, myocardial infarction, and congestive heart failure, suggesting that DR may be an important predictive factor for evaluating adverse cardiovascular outcomes. Currently, there are limited physiological explanations for the direct link between retinal lesions in individuals with diabetes and the risk of CVD. Reaven et al. proposed in a cross-sectional study that DR, as a microvascular lesion, indicates hemodynamic abnormalities that lead to platelet aggregation, increased blood viscosity, and arterial sclerosis, which further induce and exacerbate the occurrence of adverse events in large blood vessels. Similar pathological changes are also present in the retinal microenvironment, which may be a common underlying mechanism for atherosclerosis and diabetic microvascular disease^41^. In addition, DR patients without obvious CVD features may also have subclinical CVD status and subclinical inflammation closely related to the degree and duration of hyperglycemia^7^. Similar to the results associated with all-cause mortality, we did not find a correlation between PDR and CVD-caused mortality. However, the SEED Study^40^ and Southern California retrospective cohort study^21^ reported opposite results, reporting a significantly higher incidence of CVD-caused mortality in DR patients compared to non-DR patients, and a significant increase in the risk of death with the severity of DR. This may be due to multiple reasons. First, considering that DM is the most important risk factor for CVD-caused mortality in patients, confounding factors such as complications related to DM and comorbidities of the vascular system may overestimate the survival risk of CVD in inadequately adjusted DR patients. Additionally, as previously described, CVD triggered by macrovascular adverse events and DR with microangiopathy as the main manifestation may enjoy the common pathophysiological mechanism, which may confound the true cause of some CVD-caused deaths. More importantly, previous studies did not consider the competing risk of CVD death, especially when estimating the mortality rate of populations over 40 years old. The use of the previous Cox proportional hazards regression model may lead to an overestimation of the absolute risk of death caused by CVD. We used a competing risk model that adjusted for multiple confounding factors, for the first time to address this methodological issue and clarify the significant relationship between DR and the mortality risk caused by CVD. Mechanistically, Steno proposed that the coincidence between PDR and CVD in diabetic subjects reflects widespread vascular disorders. It is speculated that the genetic polymorphism of enzymes involved in heparan sulfate proteoglycan metabolism present in the mesangium, retina, and endothelium of large blood vessels may be responsible for the symptoms of proteinuria and related complications, including DR, in individuals with diabetes. These changes lead to the progression of albuminuria, mesangial expansion, retinal lesions, and large blood vessel lesions^42^.

It is worth noting that, after multiple adjustments for confounding factors or PSM, the presence of DR is still correlated with increased DM-caused mortality. Severity analysis results confirmed that, unlike all-cause mortality and CVD-caused mortality, various grades of DR, including PDR, are associated with DM-caused mortality. In addition, the DM exacerbation trend was significantly associated with worsening survival in DM patients, with the corresponding effect values all significantly higher than the risk of CVD-caused mortality and other factors. Most studies support our research results, such as the SEED Study^40^, the EURODIAB Prospective Complications Study^38^, and the Singapore Malay Eye Study^43^, which are based on large-scale population cohorts. However, the results of a study focusing on predictive factors for mortality in patients with type 2 diabetes showed that the death risk was significantly lower in DR patients alone than in those with concomitant diabetic kidney disease (DKD) or CVD. Therefore, they believed that other complications related to DR may play a joint or even a major role in affecting the DM-caused mortality^36^. The Susceptibility-Reykjavik Study also showed that the presence of vascular comorbidities such as CVD and chronic kidney disease (CKD), as well as cognitive burden and neurological dysfunction, have synergistic effects and adverse impacts on the long-term survival of patients with retinal lesions^44^. Considering that elderly people with long-term diabetes often have other systemic diseases, including CKD and CVD, the combined effects of DM-related complications have been reported to significantly increase the risk of death in DM patients. This may be the reason why we found that the DM-caused mortality is several times higher than the CVD-caused mortality and other causes in our study^45^. On the other hand, the severity analysis suggested that, compared to patients without DR, the DM-caused mortality increases exponentially as DR grade increases, especially in PDR, where the DM-caused mortality is more than 13 times higher than in patients without DR. The results after PSM also show that the DM-caused mortality in diabetes participants with any degree of DR is more than five times higher than patients without DR. Considering that retinopathy has been reported as an important indicator of the development stage of DM, our results confirm the existence and severity of DR may become a strong predictor of survival in DM patients. Given the convenience and accuracy of retinal examination in clinical practice, our results suggest that early retinal information and close monitoring can avoid vascular accumulation damage caused by time, and may provide valuable clinical reference for regular screening and future prognosis assessment in the early DM diagnosis^21^.

In summary, as previously described, the retina, as the only part of the body where blood vessels can be directly observed, may be an effective predictor of survival risk for individuals with diabetes through retina imaging screening and grading diagnosis of DR. The use of multifactor-adjusted Fine and Gray competing risk regression models and PSM methods allowed us to clarify the relationship between DR and the increased risk of CVD, as well as DM caused mortality. Our study has several advantages. Firstly, we used a large-scale nationally representative sample of non-hospitalized elderly individuals with diabetes aged 40 and above in the United States and a standardized DR grading assessment method. Secondly, we used a multifactor-adjusted Fine and Gray competing risk regression models to explore the relationship between DR and special-cause mortality. In addition, previous studies have ignored important confounding factors such as CVD and age-related ocular comorbidities, whereas we have fully accounted for these confounding factors and further used the PSM approach to fully balance these features. Finally, we also conducted the trend test of DR severity grading and mortality to further explore the dynamic changes in survival risk for individuals with diabetes with DR.

However, this study also has some limitations. Firstly, the diagnosis of DR and the assessment of confounding factors were both carried out at the same time, and changes in patients' health behaviors and comorbidities, including CVD and DM complications, during the follow-up period were unknown. Secondly, although we tried to include a range of potential confounders, some important confounders, such as eye surgery and drug use, were unable to be included since they were difficult to assess. Thirdly, information from hospitalized patients was not recorded, which may result in missing some severe cases, and the application of questionnaires and interview data may also lead to response bias.

## Conclusions

Overall, we have confirmed in this nationally representative sample of elderly non-hospitalized diabetes participants in the United States that DR is associated with increased all-cause, CVD-caused, and DM-caused mortality. Severity analysis of DR showed that mild and moderate to severe NPDR are associated with increased all-cause and CVD-caused mortality, while various grades of DR, including PDR, are related to DM-caused mortality. Compared with patients without DR, the risk of DM-caused mortality increases exponentially with increasing DR severity. The use of multiple confounding factor-adjusted competing risk regression models and PSM methods clarifies the relationship between DR and increased mortality risk due to CVD and DM, emphasizing that DR grading may serve as an effective predictive indicator for continuous monitoring of vascular status in diabetes patients and could have significant value in regular screening and future prognostic assessment.

### Supplementary Information

Supplementary Information.

## Data Availability

Publicly available datasets were analyzed in this study. This data can be found here: https://www.cdc.gov/nchs/nhanes/index.htm.
