# Supplementary material for: Association of diabetic retinopathy on all-cause and cause-specific mortality in older adults with diabetes: National Health and Nutrition Examination Survey, 2005–2008
Source: Sci Rep. 2024 May 7;14:10458. doi: 10.1038/s41598-024-58502-z (PMC11076637; doi:10.1038/s41598-024-58502-z)
Supplement: Supplementary file 1 — Supplementary Information. [file 41598_2024_58502_MOESM1_ESM.docx]

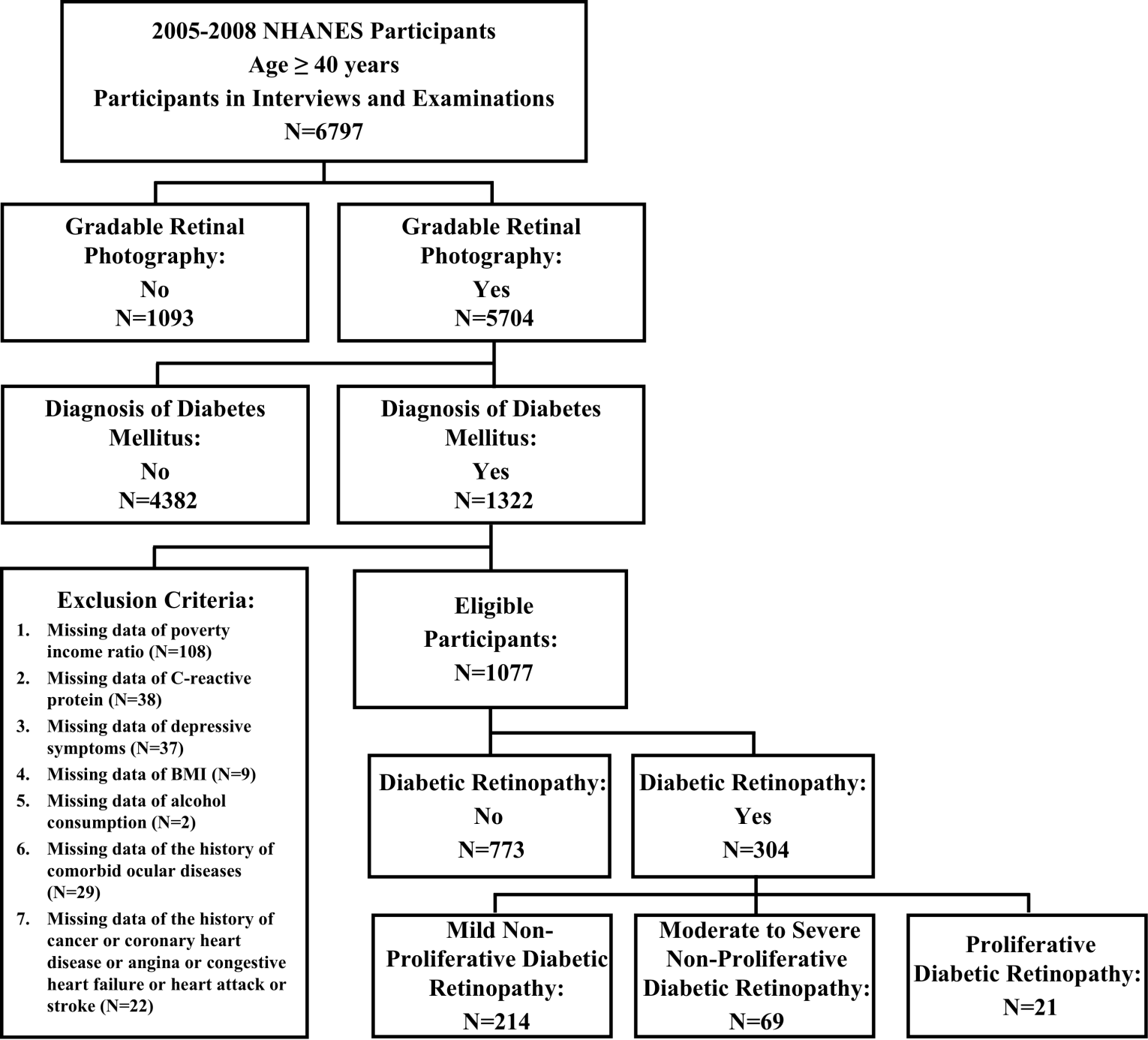


**Figure S1.** Schematic Showing Inclusion Criteria for Study Participants. Schematic showing study participants included for the present analysis from the 2005-2008 National Health and Nutrition Examination Survey(NHANES). A total of 1077 participants were included.


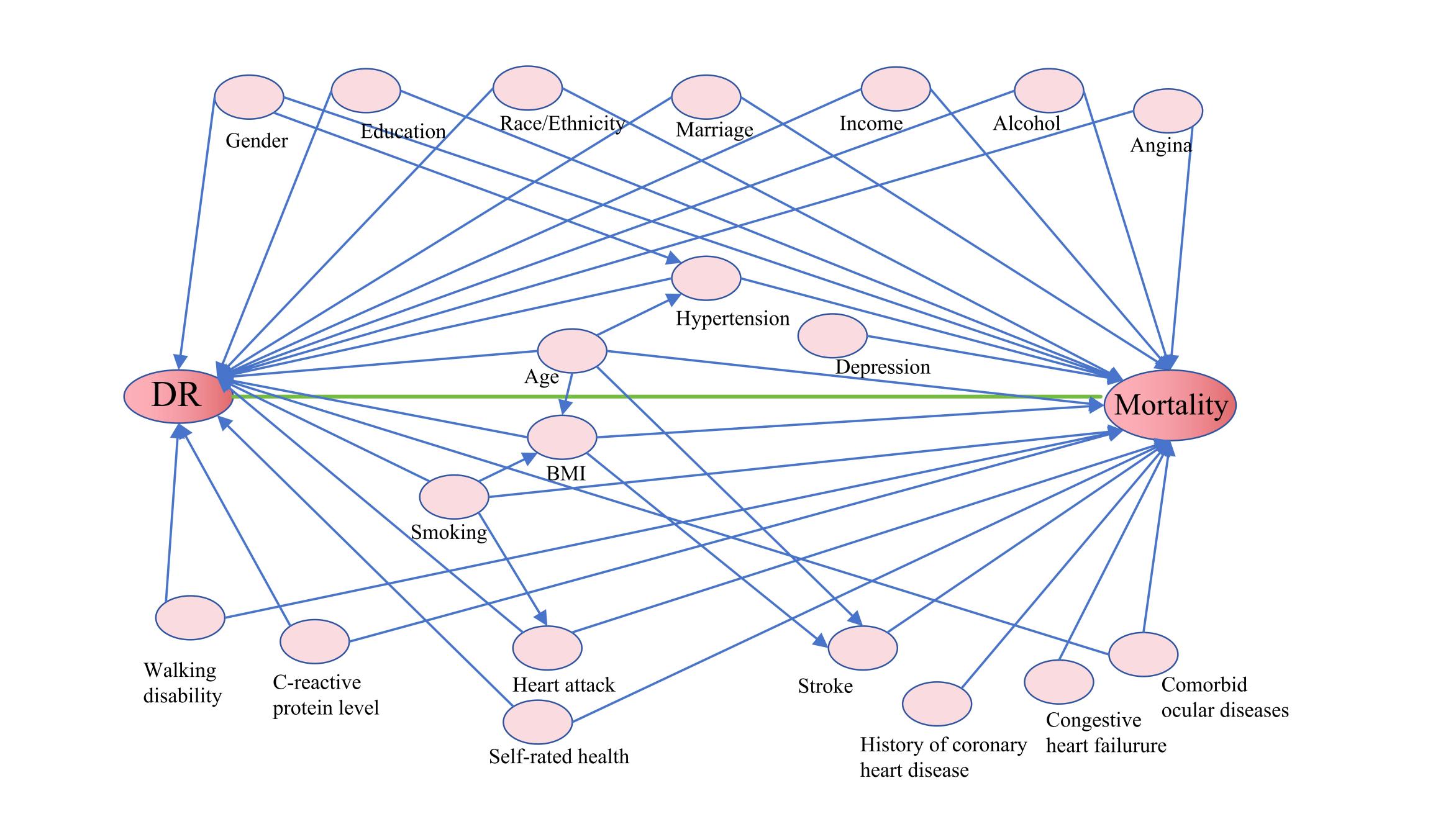


**Figure S2.** Directed Acyclic Graph for Covariates. A total of 20 covariates were include.

**Table S1. Demographic, Health Behavior, and General Health Characteristics of Participants by Diabetic Retinopathy Status after PSM ^a^**

| **Characteristic** | **Study Participants after PSM** | | | |
| --- | --- | --- | --- | --- |
|  | **All (n = 608)**  **N = 543,326.5** | **No Diabetic Retinopathy (n = 304)**  **N = 277,206.9** | **Diabetic Retinopathy (n = 304)**  **N = 266,119.6** | ***P* value** |
| Age, No. (%), y |  |  |  | 0.98 |
| 40-49 | 65 (10.69) | 30 (16.53) | 35 (16.44) |  |
| 50-59 | 125 (20.56) | 61 (26.57) | 64 (26.16) |  |
| 60-69 | 217 (35.69) | 109 (27.87) | 108 (27.16) |  |
| 70-79 | 150 (24.67) | 78 (22.26) | 72 (21.98) |  |
| ≥80 | 51 (8.39) | 26 (6.77) | 25 (8.26) |  |
| Sex, No. (%) |  |  |  | 0.26 |
| Male | 348 (57.24) | 176 (54.23) | 172 (57.88) |  |
| Female | 260 (42.76) | 128 (45.77) | 132 (42.12) |  |
| Race/Ethnicity, No. (%) |  |  |  | 0.46 |
| Non-Hispanic White | 254 (41.78) | 135 (67.41) | 119 (67.01) |  |
| Non-Hispanic Black | 186 (30.59) | 89 (16.52) | 97 (19.23) |  |
| Mexican American | 117 (19.24) | 57 (7.08) | 60 (8.05) |  |
| Other | 51 (8.39) | 23 (8.99) | 28 (5.71) |  |
| Marital status, No. (%) |  |  |  | 0.55 |
| Unmarried or other | 231 (37.99) | 111 (32.22) | 120 (34.71) |  |
| Married or living with a partner | 377 (62.01) | 193 (67.78) | 184 (65.29) |  |
| Educational attainment, No. (%) |  |  |  | 0.99 |
| < High school | 269 (44.24) | 134 (31.49) | 135 (31.43) |  |
| ≥High school | 339 (55.76) | 170 (68.51) | 169 (68.57) |  |
| Poverty income ratio, No. (%) |  |  |  | 0.42 |
| Below poverty line (<1.00) | 113 (18.59) | 56 (10.56) | 57 (12.43) |  |
| At or above poverty line (≥1.00) | 495 (81.41) | 248 (89.44) | 247 (87.57) |  |
| Alcohol consumption, No. (%) |  |  |  | 0.7 |
| Never | 110 (18.09) | 53 (17.75) | 57 (19.55) |  |
| Former | 238 (39.14) | 116 (38.81) | 122 (36.40) |  |
| Mild | 175 (28.78) | 96 (30.52) | 79 (31.49) |  |
| Moderate | 44 (7.24) | 20 (9.11) | 24 (6.53) |  |
| Heavy | 41 (6.74) | 19 (3.82) | 22 (6.03) |  |
| BMI, No. (%) |  |  |  | 0.5 |
| 18.5-30.0 | 283 (46.55) | 138 (45.33) | 145 (40.51) |  |
| <18.5 | 2 (0.33) | 1 (0.33) | 1 (0.13) |  |
| ≥30.0 | 323 (53.12) | 165 (54.34) | 158 (59.36) |  |
| High C-reactive protein level, No. (%) |  |  |  | 0.5 |
| No | 524 (86.18) | 265 (82.81) | 259 (85.05) |  |
| Yes | 84 (13.82) | 39 (17.19) | 45 (14.95) |  |
| Hypertension, No. (%) |  |  |  | 0.67 |
| No | 154 (25.33) | 89 (30.19) | 65 (27.74) |  |
| Yes | 454 (74.67) | 215 (69.81) | 239 (72.26) |  |
| Hyperlipidemia, No. (%) |  |  |  | 0.85 |
| No | 75 (12.34) | 39 (10.94) | 36 (11.50) |  |
| Yes | 533 (87.66) | 265 (89.06) | 268 (88.50) |  |
| Depressive symptoms, No. (%) |  |  |  | 0.54 |
| No | 557 (91.61) | 277 (90.53) | 280 (91.86) |  |
| Yes | 51 (8.39) | 27 (9.47) | 24 (8.14) |  |
| Difficulty walking, No. (%) |  |  |  | 0.99 |
| No | 492 (80.92) | 247 (79.92) | 245 (79.96) |  |
| Yes | 116 (19.08) | 57 (20.08) | 59 (20.04) |  |
| Health status, No. (%) |  |  |  | 0.78 |
| Poor to fair | 277 (45.56) | 129 (38.04) | 148 (39.34) |  |
| Good to excellent | 331 (54.44) | 175 (61.96) | 156 (60.66) |  |
| History of congestive heart failure, No. (%) |  |  |  | 0.43 |
| No | 525 (86.35) | 268 (87.80) | 257 (85.11) |  |
| Yes | 83 (13.65) | 36 (12.20) | 47 (14.89) |  |
| History of coronary heart disease, No. (%) |  |  |  | 0.76 |
| No | 538 (88.49) | 273 (87.10) | 265 (86.02) |  |
| Yes | 70 (11.51) | 31 (12.90) | 39 (13.98) |  |
| History of angina, No. (%) |  |  |  | 0.9 |
| No | 563 (92.6) | 284 (91.15) | 279 (91.58) |  |
| Yes | 45 (7.4) | 20 (8.85) | 25 (8.42) |  |
| History of heart attack, No. (%) |  |  |  | 0.71 |
| No | 535 (87.99) | 274 (88.82) | 261 (87.67) |  |
| Yes | 73 (12.01) | 30 (11.18) | 43 (12.33) |  |
| History of stroke, No. (%) |  |  |  | 0.84 |
| No | 537 (88.32) | 273 (87.54) | 264 (86.74) |  |
| Yes | 71 (11.68) | 31 (12.46) | 40 (13.26) |  |
| History of cancer, No. (%) |  |  |  | 0.84 |
| No | 524 (86.18) | 261 (86.44) | 263 (87.13) |  |
| Yes | 84 (13.82) | 43 (13.56) | 41 (12.87) |  |
| History of comorbid ocular diseases, No. (%) |  |  |  | 0.54 |
| No | 364 (59.87) | 184 (64.19) | 180 (60.95) |  |
| Yes | 244 (40.13) | 120 (35.81) | 124 (39.05) |  |

Abbreviations: NPDR, Non-proliferative diabetic retinopathy; PDR, proliferative diabetic retinopathy; PSM, Propensity score matching; BMI, body mass index (calculated as weight in kilograms divided by height in meters squared).

^a^ All proportions, means, and SEs are weighted estimates of the US population characteristics, taking into account the complex sampling design of the National Health and Nutrition Examination Survey.

**Table S2. Demographic, Health-Related Behaviors and General Health Characteristics of Participants Included and Excluded in the Analyses ^a^**

| **Characteristic** | **No. of Excluded**  **Subjects (n = 245)** | **No. of Included**  **Subjects (n = 1077)** | ***P* value ^b^** |
| --- | --- | --- | --- |
| Age, No. (%), y |  |  | **< 0.0001** |
| 40-49 | 23 (14.02) | 136 (18.52) |  |
| 50-59 | 37 (17.04) | 255 (29.81) |  |
| 60-69 | 83 (27.31) | 365 (27.26) |  |
| 70-79 | 64 (26.71) | 240 (18.35) |  |
| ≥80 | 38 (14.92) | 81 (6.06) |  |
| Sex, No. (%) |  |  | **0.004** |
| Male | 106 (36.38) | 559 (50.76) |  |
| Female | 139 (63.62) | 518 (49.24) |  |
| Race/Ethnicity, No. (%) |  |  | **0.001** |
| Non-Hispanic White | 79 (53.82) | 484 (70.73) |  |
| Non-Hispanic Black | 77 (21.53) | 283 (14.23) |  |
| Mexican American | 56 (10.38) | 200 ( 7.23) |  |
| Other | 33 (14.28) | 110 ( 7.81) |  |
| Marital status, No. (%) |  |  | 0.52 |
| Unmarried or other | 96 (35.32) | 413 (32.76) |  |
| Married or living with a partner | 149 (64.68) | 664 (67.24) |  |
| Educational attainment, No. (%) |  |  | **0.05** |
| < High school | 112 (34.23) | 414 (25.08) |  |
| ≥High school | 133 (65.77) | 663 (74.92) |  |
| Poverty income ratio, No. (%) |  |  | **0.001** |
| Below poverty line (<1.00) | 37 (22.90) | 209 (11.70) |  |
| At or above poverty line (≥1.00) | 100 (77.10) | 868 (88.30) |  |
| Alcohol consumption, No. (%) |  |  | 0.11 |
| Never | 45 (23.37) | 185 (16.72) |  |
| Former | 79 (34.70) | 382 (32.03) |  |
| Mild | 44 (23.20) | 306 (31.10) |  |
| Moderate | 19 ( 8.04) | 109 (12.58) |  |
| Heavy | 19 (10.70) | 95 ( 7.57) |  |
| BMI, No. (%) |  |  | **0.02** |
| 18.5-30.0 | 103 (49.86) | 465 (38.74) |  |
| <18.5 | 1 (1.76) | 5 (0.25) |  |
| ≥30.0 | 125 (48.38) | 607 (61.00) |  |
| High C-reactive protein level, No. (%) |  |  | 0.44 |
| No | 168 (83.85) | 884 (80.55) |  |
| Yes | 34 (16.15) | 193 (19.45) |  |
| Hypertension, No. (%) |  |  | 0.75 |
| No | 63 (29.70) | 279 (28.08) |  |
| Yes | 182 (70.30) | 798 (71.92) |  |
| Hyperlipidemia, No. (%) |  |  | 0.07 |
| No | 43 (17.09) | 128 (11.26) |  |
| Yes | 202 (82.91) | 949 (88.74) |  |
| Depressive symptoms, No. (%) |  |  | 0.09 |
| No | 163 (85.43) | 963 (89.91) |  |
| Yes | 31 (14.57) | 114 (10.09) |  |
| Difficulty walking, No. (%) |  |  | **0.002** |
| No | 173 (71.60) | 900 (84.21) |  |
| Yes | 72 (28.40) | 177 (15.79) |  |
| Health status, No. (%) |  |  | **0.01** |
| Poor to fair | 135 (48.87) | 463 (35.32) |  |
| Good to excellent | 110 (51.13) | 614 (64.68) |  |
| History of congestive heart failure, No. (%) |  |  | **0.01** |
| No | 200 (86.25) | 982 (92.03) |  |
| Yes | 35 (13.75) | 95 (7.97) |  |
| History of coronary heart disease, No. (%) |  |  | 0.92 |
| No | 206 (88.85) | 963 (88.61) |  |
| Yes | 31 (11.15) | 114 (11.39) |  |
| History of angina, No. (%) |  |  | **0.04** |
| No | 206 (87.24) | 1006 (93.29) |  |
| Yes | 29 (12.76) | 71 (6.71) |  |
| History of heart attack, No. (%) |  |  | **0.01** |
| No | 195 (81.88) | 962 (89.66) |  |
| Yes | 48 (18.12) | 115 (10.34) |  |
| History of stroke, No. (%) |  |  | **0.001** |
| No | 194 (80.77) | 975 (90.78) |  |
| Yes | 46 (19.23) | 102 ( 9.22) |  |
| History of cancer, No. (%) |  |  | 0.09 |
| No | 209 (80.86) | 927 (85.70) |  |
| Yes | 34 (19.14) | 150 (14.30) |  |
| History of comorbid ocular diseases, No. (%) |  |  | 0.15 |
| No | 128 (63.26) | 718 (69.86) |  |
| Yes | 82 (36.74) | 359 (30.14) |  |
| Mortality leading |  |  | **0.003** |
| Cancer-Specific | 17 (5.05) | 77 (5.77) |  |
| CVD-Specific | 42 (17.02) | 140 (11.66) |  |
| DM-Specific | 11 (3.39) | 35 (3.07) |  |
| Other | 46 (20.71) | 127 (10.56) |  |
| No | 129 (53.82) | 698 (68.93) |  |
| Mortality status |  |  | **0.004** |
| Assumed alive | 129 (53.82) | 698 (68.93) |  |
| Assumed deceased | 116 (46.18) | 379 (31.07) |  |
| DR status |  |  |  |
| None | 164 (70.55) | 773 (75.29) |  |
| Any DR | 81 (29.45) | 304 (24.71) | 0.22 |
| Mild NPDR | 45 (18.21) | 214 (18.11) | 0.06 |
| Moderate to Severe NPDR | 26 (7.90) | 69 (5.39) |  |
| PDR | 10 (3.33) | 21 (1.21) |  |

Abbreviations: NPDR, Non-proliferative diabetic retinopathy; PDR, proliferative diabetic retinopathy; BMI, body mass index (calculated as weight in kilograms divided by height in meters squared).

^a^ Mortality was assessed through December 31, 2020. All proportions, means, and SEs are weighted estimates of the US population characteristics, taking into account the complex sampling design of the National Health and Nutrition Examination Survey.

^b^ All *P* values were calculated using the unpaired t test for continuous variables and the design-adjusted Rao-Scott Pearson χ^2^ test for categorical variables. Comparisons were between each group with retinopathy and the group with no retinopathy and were unadjusted.

^*^ *P*<0.05; ^**^ *P*<0.01; ^***^ *P*<0.001; ^****^ *P*<0.0001.

**Table S3. Cox Proportional Hazards Models for All-Cause Mortality and Fine and Gray Competing Risks Regression Models for Specific-Cause Mortality by Diabetic Retinopathy Status after PSM**

| **Retinopathy Status** | **Mortality** | | | | |
| --- | --- | --- | --- | --- | --- |
|  | **sHR (95% CI)** | | | | |
|  | **Due to all causes**  **n = 242**  **N = 210,503.2** | **Due to cancer**  **n = 50**  **N = 41,431.48** | **Due to CVD**  **n = 91**  **N = 82,193.42** | **Due to DM**  **n = 24**  **N = 20,010.11** | **Due to other causes**  **n = 77**  **N = 66,868.16** |
| **No Diabetic Retinopathy (n = 304)**  **N = 277,206.9** | 1 [Reference]  n = 106  N = 174,215.6 | 1 [Reference]  n = 30  N = 27,134.01 | 1 [Reference]  n = 36  N = 32,557.78 | 1 [Reference]  n = 6  N = 3,391.504 | 1 [Reference]  n = 34  N = 30,580.59 |
| **Diabetic Retinopathy (n = 304)**  **N = 266,119.6** | 1.35 (1.03 to 1.78)^*^  n = 136  N = 179,922.6 | 0.66 (0.32 to 1.34)  n = 20  N = 14,297.47 | 1.71 (1.07 to 2.73)^*^  n = 55  N = 49,635.65 | 5.56 (2.28 to 13.59)^***^  n = 18  N = 16,618.6 | 1.40 (0.85 to 2.31)  n = 43  N = 36,287.57 |
| ***P* for trend** | 0.03 | 0.25 | 0.03 | <0.001 | 0.18 |

Abbreviations: CVD, cardiovascular disease; DM, Diabetes mellitus; PSM, Propensity score matching; sHR, subdistribution hazard ratio.

^*^ *P*<0.05; ^**^ *P*<0.01; ^***^ *P*<0.001; ^****^ *P*<0.0001.
